# Analytical validation and initial clinical testing of quantitative microscopic evaluation for PD-L1 and HLA I expression on circulating tumor cells from patients with non-small cell lung cancer

**DOI:** 10.1186/s40364-022-00370-8

**Published:** 2022-04-25

**Authors:** Jennifer L. Schehr, Nan Sethakorn, Zachery D. Schultz, Camila I. Hernandez, Rory M. Bade, Diego Eyzaguirre, Anupama Singh, David J. Niles, Leslie Henderson, Jay W. Warrick, Scott M. Berry, Kaitlin E. Sundling, David J. Beebe, Ticiana A. Leal, Joshua M. Lang

**Affiliations:** 1 Carbone Cancer Center, University of Wisconsin, Madison, WI USA; 2 Department of Medicine, University of Wisconsin, Madison, WI USA; 3 Department of Biomedical Engineering, University of Wisconsin, Madison, WI 53705 USA; 4 Wisconsin State Lab of Hygiene, Madison, WI USA; 5 Department of Pathology and Laboratory Medicine, University of Wisconsin, Madison, WI USA; 6 Department of Medicine, Carbone Cancer Center, University of Wisconsin, 1111 Highland Avenue, WIMR 7151, Madison, WI 53705 USA

**Keywords:** Circulating tumor cell, Non-small cell lung Cancer, Immunotherapy biomarker, Precision medicine

## Abstract

**Introduction:**

PD-L1 expression in non-small cell lung cancer (NSCLC) predicts response to immune checkpoint blockade, however is an imperfect biomarker given tumor heterogeneity, and the antigen presentation pathway requiring other components including HLA I expression. HLA I downregulation may contribute to resistance, warranting its evaluation in attempts to guide patient selection. In addition, earlier detection of acquired resistance could prompt earlier change in treatment and prolong patient survival. Analysis of circulating tumor cells (CTCs) captures heterogeneity across multiple sites of metastases, enables detection of changes in tumor burden that precede radiographic response, and can be obtained in serial fashion.

**Methods:**

To quantify the expression of both PD-L1 and HLA I on CTCs, we developed exclusion-based sample preparation technology, achieving high-yield with gentle magnetic movement of antibody-labeled cells through virtual barriers of surface tension. To achieve clinical-grade quantification of rare cells, we employ high quality fluorescence microscopy image acquisition and automated image analysis together termed quantitative microscopy.

**Results:**

In preparation for clinical laboratory implementation, we demonstrate high precision and accuracy of these methodologies using a diverse set of control materials. Preliminary testing of CTCs isolated from patients with NSCLC demonstrate heterogeneity in PD-L1 and HLA I expression and promising clinical value in predicting PFS in response to PD-L1 targeted therapies.

**Conclusions:**

By confirming high performance, we ensure compatibility for clinical laboratory implementation and future application to better predict and detect resistance to PD-L1 targeted therapy in patients with NSCLC.

**Supplementary Information:**

The online version contains supplementary material available at 10.1186/s40364-022-00370-8.

## Background

Stage IV non-small cell lung cancer (NSCLC) is incurable with 5-year survival rates of less than 5% with platinum doublet chemotherapy, however, targeting PD-1/PD-L1 improved overall survival (OS) up to 30 [1]. Immunotherapy via blockade of the programmed death ligand 1 (PD-L1) pathway aims to counteract immune evasion by shifting the balance in favor of immune activation, enabling T cell mediated cytotoxicity. However, only a subset of patients benefit from these often toxic therapeutic approaches, [2] requiring diagnostic tools which can better predict response. Patients with high PD-L1 experience better outcomes but nearly 50% of PD-L1 high patients progressed on pembrolizumab within the first year, [3] suggesting additional resistance mechanisms occur in vivo. Currently, PD-L1 expression is evaluated by immunohistochemical analysis of tissue biopsies, however the sensitivity and specificity associated with these methods are limited due to temporal and spatial heterogeneity in PD-L1 expression [4–8]. For example, the concordance of PD-L1 expression between paired primary tumors and metastatic lymph nodes was approximately 30% using the two main clinically used antibodies (Dako 22C3 and Dako 28–8) [6]. Patients with NSCLC undergoing neoadjuvant chemotherapy or combination chemoradiotherapy prior to curative-intent surgery demonstrated that 36–57% of patients had either an increase or decrease in PD-L1 expression in their post-treatment specimen compared to the pre-treatment diagnostic biopsy [9–12]. Similarly, patients undergoing neoadjuvant anti-PD-L1/PD-1 therapy demonstrated no correlation between pre-treatment and post-surgical PD-L1 expression on tumor and/or immune cells [13]. An autopsy study of two patients with hyperprogression on pembrolizumab showed that both patients had rapid downregulation of PD-L1 [14]. Many patients who respond initially developed acquired resistance, the mechanisms of which remain poorly understood due in part to the challenges in obtaining repeat biopsies. Advances in precision medicine will be achieved not only through more complex combinations of therapies, but also with increasingly more sophisticated diagnostic tools including longitudinal analysis of molecular changes that occur throughout the course of therapy.

A critical step to neoantigen presentation and T cell response involves the presentation of intra-cellular peptides on the cell surface for recognition by T cell receptors through human leukocyte antigen class I (HLA I). HLA I is required for tumor cell lysis by cytotoxic T cells, [15] and its downregulation is observed in 49% of lung carcinomas [16]. This phenotypic manifestation may involve genotypic alterations as loss of heterozygosity (LOH) in the HLA locus occurs in 40% of patients with NSCLC [15]. Thus, aberrant neoantigen presentation could be an important mechanism of resistance to PD-L1-targeted therapy. Recent studies have suggested that HLA expression correlates with response to immunotherapy, and predictive power interacts with other biomarkers. Higher HLA expression was associated with increased CD8+ T cell infiltration in NSCLC, [16] LOH in at least one HLA I locus correlated with poor progression-free and overall survival in patients with NSCLC being treated with checkpoint inhibitors [17, 18]. One prospective study demonstrated interactions between HLA expression and other biomarkers such as tumor mutational load, PD-L1 expression, and infiltrating CD8 T cells as predictors of response to single-agent nivolumab in NSCLC, but no difference when stratified by HLA loss alone [19]. Similarly, the positive prognostic effect of CD8 T cells was only observed in patients with high expression of HLA I, [20] and correction for HLA LOH improved the predictive utility of tumor mutation burden in response to PD1/PD-L1 blockade [21]. These studies indicate that moving beyond tumor PD-L1 expression as a biomarker of response is key to understanding resistance to immunotherapies.

Tumor cell expression of PD-L1 and HLA I may fluctuate over the course of therapy, introducing a need for liquid biopsy approaches to biomarker testing. For the purpose of early identification of intrinsic and acquired resistance to anti-cancer therapies, we have developed a minimally invasive diagnostic test using circulating tumor cells (CTCs) captured and analyzed from whole blood using exclusion-based sample preparation (ESP) technology [22–25]. ESP leverages microfluidics to reduce sample loss for high yield retention of rare cell targets, which has now evolved from a manual device into a fully automated system with the development of the ExtractMax from Gilson and Salus, poising it for success in clinical diagnostic implementation [26–28]. To enable objective quantification of protein expression on rare cells, high-quality image acquisition maintaining uniform focus is combined with automated image analysis algorithms for quantitative microscopy.

Translation of basic research methods for diagnostic implementation requires fulfillment of regulatory guidelines per Clinical Laboratory Improvement Amendments (CLIA), including analytical validation of a method’s accuracy, precision, analytical specificity and analytical sensitivity. The CLIA regulations ensure that clinical decisions are based on reliable test results to ultimately protect patients, where methods must demonstrate and be continually monitored for accuracy, precision, antibody specificity, and extraction efficiency. Using multiple experimental systems, we demonstrate the analytical validity and control systems capable of monitoring the performance of ESP-quantitative microscopy for evaluating PD-L1 and HLA I expression on CTCs from patients with NSCLC with the potential to better predict and detect resistance to PD-L1 targeted therapy.

## Methods

### Cell lines

Cell lines (H358 and LNCaP) were obtained from ATCC and maintained in cell culture flasks in RPMI 1640 (Corning, Corning, NY) with 10% FBS (Life Technologies, Carlsbad, CA) and 2% penicillin/streptomycin (Hyclone, Logan, Utah) at 37 C with 5% CO_2_. Cell lines were authenticated by short tandem repeat profiling in January of 2021 by the UW-Madison TRIP lab. To differentiate the different cell types from each other in the cell line mixing experiment (Supplemental Fig. 3), LNCaPs were stained with Calcein AM (Life Technologies) at 37 C for 15 min, then washed once with PBS (Life Technologies) prior to mixing.

### Polymer beads

Uniformly fluorescent calibration beads (Bangs Laboratories, Fishers, IN) were used to evaluate the precision of the quantitative microscopy. ELISA beads, polymer beads coated with recombinant protein, were used to evaluate accuracy. Similar to a sandwich ELISA, reactive polymer beads (Bangs Laboratories) were labeled with antibodies against PD-L1 (Biolegend, 29E.2A3) (per manufacturer’s guidelines), then incubated with recombinant human PD-L1 (Biolegend, San Diego, CA) of varying quantities for 20 min at room temperature (RT) in PBS, then stained with fluorescent antibodies against PD-L1 (eBiosciences, San Diego, CA) in 10% FBS (in PBS) for 20 min at RT. Following this same method, HLA I ELISA beads were generated by capturing and detecting recombinant human HLA I (Abnova, Taipei, Taiwan) with antibodies from the matched ELISA kit (Abnova). By varying the amount of protein standard loaded onto the surface of the bead, these synthetic samples mimic different quantities of proteins expressed on the surface of a cell. The polymer beads are internally dyed with Glacial Blue which fluoresces at a wavelength similar to that of Hoechst, further mimicking the cell staining and enabling downstream image analysis algorithms used to evaluate CTCs in patient samples.

### Patient samples

This was a prospective study to evaluate quantification of PD-L1 and HLA I in circulating tumor cells from patients with metastatic NSCLC, (*n* = 22 total patients, *n* = 17 patients who had ever received chemo/immunotherapy or immunotherapy alone, *n* = 14 patients who received immunotherapy and had matched baseline CTC assessment (baseline CTC analysis obtained within 3 weeks of starting immunotherapy), *n* = 12 patients who received immunotherapy, had matched baseline CTC analysis, and also had matched baseline PD-L1 tissue biopsy assessment with the FDA-approved PD-L1 22C3 PharmDx Dako assay. Patients were consented under IRB-approved protocol (2014–1214) prior to any study procedures. To harvest PBMCs, 50 mL of blood was drawn by venipuncture into 10 mL EDTA vacutainer tubes, each 15 mL volume of blood was diluted 1:1 with PBS (Life Technologies), underlaid with 10 mL of ficoll-paque (General Electric, Boston, MA), and spun for 20 min at 980 g in a swing-bucket rotor centrifuge. PBMCs were washed twice with PBS and then depleted of CD45+ cells using a standard LS MACS column (Miltenyi, Bergisch Gladbach, Germany) per manufacturer’s guidelines.

### ESP cell capture and staining

To facilitate magnetic capture and manipulation of target cells, Streptavidin-blocked SeraMag SpeedBead Paramagnetic Particles (PMPs) (General Electric) were conjugated to antibodies against EpCAM (R&D Systems, Minneapolis, MN), MUC1 clone 16A (Biolegend), and TROP-2 (R&D) by first washing the PMPs twice with 0.1% PBST in PBS then adding 1 uL of each antibody previously conjugated to DSB-X biotin per manufacturer’s guidelines from the Dynabeads FlowComp Flexi Kit (Thermo Fisher Scientific, Waltham, MA). After incubating 20 min at room temperature with constant vortex agitation, beads were washed twice with 10% FBS in PBS, then allowed to bind to cell line cells or CD45-depleted patient sample PBMCs for 20 min under constant rotation at 4 C in a total volume of 500 uL of 10% FBS. For automated sample processing, cells bound to PMPs were transferred to the input wells of the ExtractMan extraction plates (100 uL final volume version) (Gilson, Middleton, WI). Cells were pulled from the input well into one large wash well (250 uL volume of 10% FBS) using the opposing magnetic forces in the hovering magnetic pipette head attachment, and the underlying magnetic bar floating within the plate platform, then mixed with slow pipette mixing to maximize viability. Cells were then pulled into a subsequent reagent well containing 1:70 concentrations in 10% FBS of extracellular fluorescent antibodies against PD-L1 clone MIH1 (eBiosciences), HLA I clone W6/32 (Biolegend), CD45 clone HI30 (Biolegend), CD34 clone 581 (Biolegend) and CD66b clone G10F5 (Biolegend), along with Hoechst 33342 pre-diluted 1:25 in 10% FBS (Life Technologies), and allowed to incubate at RT for 20 min. Cells were then pulled into BD fix/perm (BD Biosciences, San Jose, CA), and incubated for 20 min at RT. Cells were then pulled into BD perm buffer containing 1:70 concentration of the intracellular fluorescent antibody against pan cytokeratin (pCK) clone C-11 previously conjugated to Alexa 790 per manufacturer’s guidelines (Biolegend) and incubated for 20 min at RT. Cells were pulled into a final well of PBS prior to imaging.

### Quantitative microscopy

For high quality imaging, suspensions of cells or polymer beads were placed into silicone isolator imaging chambers (Electron Microscopy Sciences, Hatfield, PA) attached to glass coverslips (Life Technologies) and pulled to the bottom of the liquid with an external magnet prior to imaging. Imaging chambers were placed into slide holder stage inserts for imaging with a Nikon Ti-e inverted fluorescence microscope (Nikon, Minato City, Japan) with an automated XYZ stage. An image was acquired of the entire area of each imaging chamber (~ 7 × 7 mm) by acquiring and stitching together smaller image tiles (~ 1 × 1 mm each) acquired at 10x magnification. To maintain uniform focus over the entire imaging area, images were acquired along a designated focus surface (FS) that was defined prior to imaging using Nikon’s Perfect Focus System (PFS). To minimize variability between imaging setups, the FS plane was defined using PFS with a uniform PFS offset (the defined distance between the automatically detected glass / air boundary and the focal plane of the cell layer) held constant between imaging setups. Images were acquired with a 10x apo objective, then analyzed with NIS Elements software (version 4.51.01) by first flatting images with rolling ball background subtraction (rolling ball size 10). A binary layer thresholding algorithm with spot detection on the 350 channel was then used to identify the area of each cell or bead. The binary layer mask was then used to calculate a mean fluorescence intensity (MFI) value for each wavelength for each individual cell or bead. The object catalog was populated with small crops of each individual cell to facilitate rapid manual review and deletion of image artifacts, if present, blinded from patient clinical details.

### Statistical analysis

Data was exported from NIS Elements into excel, then graphed as dot plots in GraphPad Prism (GraphPad Software, San Diego, CA) to facilitate population clustering similar to the interpretation of flow cytometry data. Log-scale axes were used to account for the log-normal distribution of fluorescence signal [29]. Refer to supplementary Fig. 1 for an example of cutoffs determined by population clustering and autofluorescence controls. Lack of spectral overlap was confirmed by acquiring and quantifying signal on calibration beads coated with each of the antibodies associated with the panel used to stain patient sample CTCs (Supplemental Fig. 2). Statistical calculations were performed on log-transformed MFI values to convert the log-normal distribution of fluorescence intensity data into a normal distribution prior to applying statistical calculations such as mean [30, 31]. The coefficient of variation (%CV) was calculated as the standard deviation divided by the mean. Significance was determined with students’ T-tests, considering *p* < 0.05 to be significant. Standard linear regression was used for assessments of linearity. Reciever operating characteristic (ROC) curve analysis was used to identify cutoffs for positivity that optimized diagnostic sensitivity and specificity for the Kaplan-Meier analysis.

## Results

**Fig. 1:**
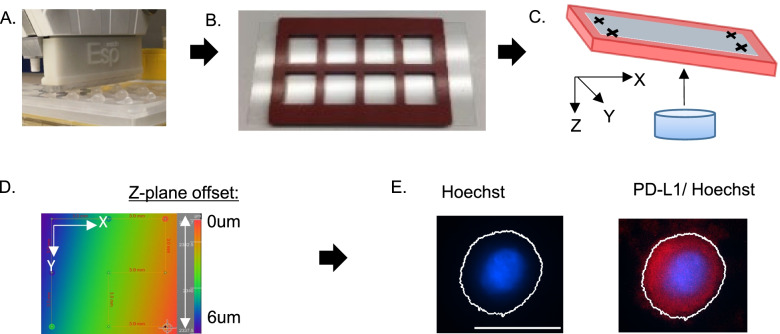
Overview of ESP-Quantitative Microscopy. **A** The ExtractMax automated platform employs ESP technology to capture and stain CTCs. **B** Glass coverslips under adhesive silicone isolators facilitate high-quality imaging of suspended cells in a total volume of 100 uL across a total area of ~ 7 × 7 mm. **C** Focus-controlled imaging accounts for variability in the focal plane due to tilt in the microscope stage across the entire imaging area. **D** The focus surface is generated by registering a Z-plane offset at each corner of the imaging area (as indicated by the heat map), enabling automated Z-plane adjustment during the acquisition of individual small image tiles (~1um each) of high magnification (10x) acquired in a grid across the entire imaging area. **E** Biomarker intensity is quantified via the generation of binary layers on the intensity of Hoechst (the 350 channel) after intensity flattening with rolling ball background subtraction using a fully automated image analysis coded “macro”

To fully evaluate all aspects of analytical performance of the assay, a combination of synthetic materials and patient samples must be employed. Isolated evaluation of earlier steps of the method (e.g., CTC capture) require the use of the final image acquisition and analysis steps and are therefore inseparable from the performance of the latter (Fig. 1). To fully evaluate the multi-step method, the reliability of the final image acquisition and analysis steps were evaluated first, building a foundation for the evaluation of upstream methodological components.

### Performance of imaging and image analysis

**Fig. 2:**
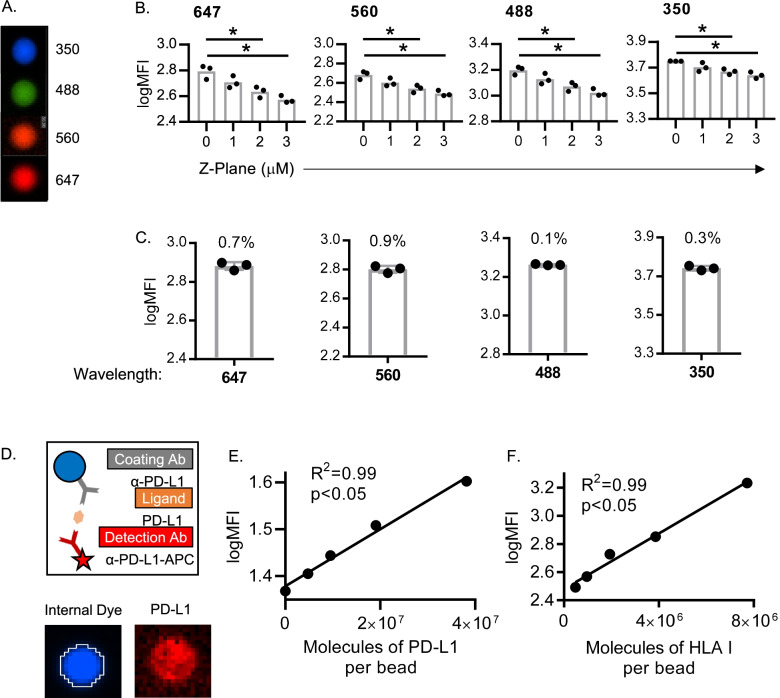
Evaluating the Precision and Accuracy of Quantitative Microscopy with Calibration Beads. **A** Example images of uniformly fluorescent spectrum calibration beads. **B** Triplicate evaluations of beads imaged at different focal planes show fluorescence intensity is significantly different at a 2 μm z-plane differential. Each symbol represents the average of the log transformed mean fluorescence intensities (MFI) of all beads within each large image (7 × 7 mm area, ~ 10,000 beads per image). **C** Beads imaged and quantified by three different analysts demonstrate the high precision of the workflows (%CV values annotated on graphs). **D** Schematic outlining how antibody-coated polymer beads (8 uM diameter) can mimic single-cells expressing different quantities of proteins, enabling an evaluation of accuracy. **E** Image of a bead showing internal dye used to define the binary layer boundary, and the fluorescence signal associated with the fluorescent anti-PD-L1 detection antibody binding to the PD-L1 ligand. **F** Serial dilution of PD-L1 ligand and **G** HLA I ligand demonstrate the accuracy of the image acquisition and analytical workflows to quantify protein expression (each symbol represents the average logMFI of all beads quantified from one large 7 × 7 mm image from each condition (~ 1000 beads per image))

Commercially available, multi-wavelength uniformly fluorescently dyed, “spectrum” calibration beads were used to evaluate the performance of the image acquisition and analysis workflows **(**Fig. 2A). Spectrum beads were pipetted into imaging chambers measuring 7 mm^2^ in area and imaged with a grid of smaller image tiles (~ 1 mm^2^) at 10x magnification, then stitched together to form one large image. Uniform focus was maintained across the entire imaging field with automated algorithms that re-focus each individual tile to account for any slight tilt in the imaging stage. The importance of maintaining uniform focus was exemplified by the acquisition of replicate large images at Z-planes differing by intervals of only 1 um (Fig. 2B). A difference of 2 um in the Z-plane led to a statistically significant difference in the quantified intensity of the fluorescence signal, highlighting the importance of uniform focus in achieving quantitative analytics.

Prior to clinical implementation, the variability of a test system must be evaluated over variables that are expected to change with normal sample processing workflows (e.g., differences in personnel, days, reagents, equipment, etc.). To evaluate the impact of these variables, images of spectrum beads were acquired and analyzed by three different analysts, on different days, using different aliquots of beads (Fig. 2C). Maintaining uniform focus between different image acquisition setups enabled high reproducibility, with %CV values all well-below 20%, the industry standard for clinical-grade precision. Spectrum bead experiments demonstrated outstanding precision in the repeatability of acquiring and analyzing images with this quantitative microscopy approach.

### Accuracy of imaging with ELISA beads

The counterpart to precision is accuracy; a method must be precise enough to obtain the same value under normal variability, but sensitive enough to detect true differences in analyte quantity. Accuracy is typically demonstrated by comparing a new method to an existing gold standard, but for methods without a comparable gold standard, test accuracy is demonstrated as linearity between results and known quantities of an analyte [32]. As a gold standard does not exist for the evaluation of PD-L1 and HLA I on CTCs, accuracy was evaluated with synthetic beads designed to mimic cells expressing different quantities of biomarkers (Fig. 2D). Similar to a sandwich ELISA, beads were coated first with a capture antibody, then with a recombinant protein standard ligand, and finally with a detection antibody. The intensity of the beads detected by the quantitative microscopy demonstrated a linear relationship to the amount of protein loaded onto the surface of the bead (R^2^ = 0.99, *p* < 0.05) (Fig. 2E and F), demonstrating the accuracy of the methodology to quantify the expression of each of these biomarkers.

### Precision and accuracy of cell staining with cell lines

**Fig. 3:**
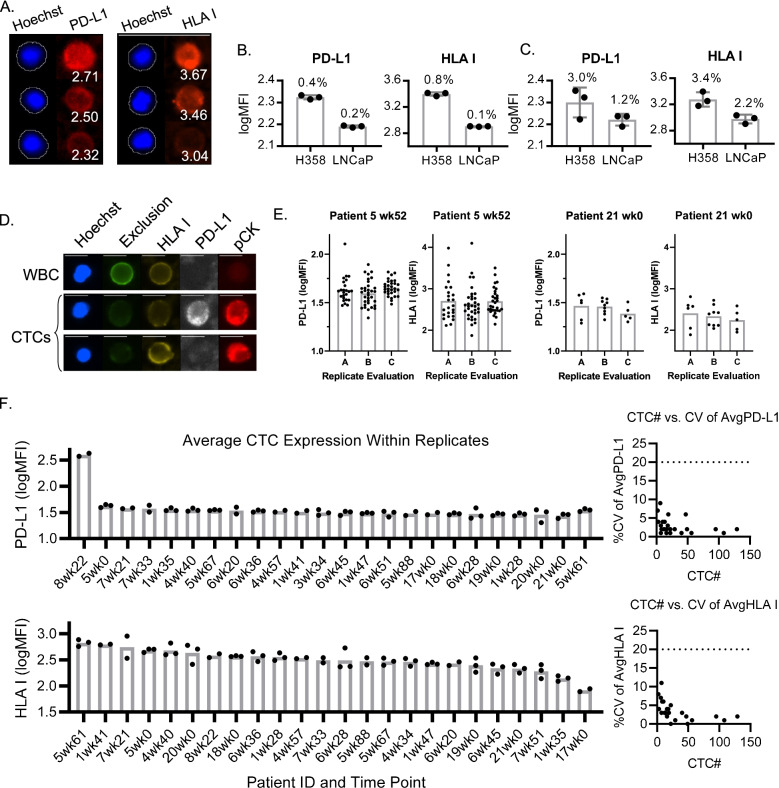
Evaluating the Precision of ESP Staining with Quantitative Microscopy with Cell Lines and Patient Samples. **A** Representative images of H358 cells with binary layers generated on Hoechst quantifying the expression of PD-L1 or HLA I. **B** Triplicate side-by-side aliquots of H358 and LNCaP cells processed with ESP technology on the ExtractMax demonstrate the high repeatability precision of the automated cell staining and quantitative microscopy workflows. Each symbol represents the average logMFI value from each condition (approximately 3000 cells per condition). %CVs of replicates are annotated on graphs. **C** Cell lines harvested from three different flasks and evaluated on three different days demonstrates high reproducibility precision. Each symbol represents the average of replicate evaluations performed on three different days. **D** Representative images of HLA I and PD-L1 staining from a WBC and CTCs from a patient sample. **E** Replicate evaluations of patient samples demonstrate high precision for patients with many CTCs (left) and with few CTCs (right). Each symbol represents a single CTC, and each column represents a replicate evaluation of a patient sample. **F** High precision is demonstrated in many patient sample replicates. Each symbol represents the average logMFI of all CTCs within one replicate. The data represents 23 samples from 11 unique patients

The confirmation of high precision and accuracy in image acquisition and analysis builds a foundation for upstream evaluations of the automated ESP cell capture and staining steps of the method. As the accuracy of cell capture was demonstrated previously (supplementary figure of Pezzi et al. [33]), here we evaluated the performance of the automated cell staining. Using ESP to capture and stain different cell lines, quantitative microscopy was able to detect different quantities of PD-L1 and HLA I (Fig. 3A). H358 cell line cells showed higher expression for both PD-L1 and HLA I biomarkers than LNCaP cell line cells, and the microscopy acquisition and analysis workflows were able to quantify the average expression of each of the cell lines with high precision in side-by-side replicates (%CVs of 0.4, 0.8, 0.2, and 0.1, respectively) (Fig. 3B). Cell lines harvested and stained from different flasks on different days also demonstrated high precision (3.0, 3.4, 1.2 and 2.2%, respectively (Fig. 3C)). The consistency to quantify both high and low levels of these biomarkers in cell lines on the same and different days demonstrated the precision of both the automated sample processing and quantitative microscopy steps across a wide range of biomarker expression levels.

As PD-L1 is more traditionally measured in tissue biopsy using a binary approach, categorizing cells as either positive or negative, we further assessed the accuracy of this test system to quantify the frequency of positively expressing cells within a mixture of different cell types (Supplemental Fig. 3). To determine the accuracy of binary quantification, H358 and LNCaP cells were mixed together at different ratios, using the results of pure population staining to predict the quantity of positive cells that would be detected in the final mixture of the different cell types. The number of both PD-L1+ and HLA I+ cells expected in each mixture showed a strong linear relationship with the number of positive cells detected by the test system (R^2^ = 0.9981 and 0.9993, respectively), demonstrating analytical accuracy for binary detection.

### Precision of the fully integrated method with patient sample replicates

Patient samples demonstrated a large range in the number of CTCs captured, ranging from 5 to 100 CTCs captured from a standard 15 mL volume of blood, [33] and precision must be demonstrated particularly for the evaluation of samples with very few CTCs. While cell line staining demonstrated high precision between replicates, each test replicate was based on a population of more than 1000 cells per condition. To ensure evaluations based on very few data points entail adequate precision, triplicate side-by-side evaluations were performed on blood drawn from individual patients (Fig. 3D and E). CD45-depleted PBMCs were split into replicates before CTC capture to evaluate the precision associated with the ESP technology, followed by quantitative microscopy.

A distribution of expression intensities was observed in the population of CTCs identified within each patient sample (Fig. 3E). Given that CTCs may reflect inter- and intra-tumoral heterogeneity [34], and the degree of CTC heterogeneity itself has been shown to be predictive of therapeutic response [35], this distribution may be due to CTC heterogeneity. As the levels of heterogeneity appear more similar within patient replicates than between different patients, this distribution can be attributed more so to CTC heterogeneity than technological variability [36].

Patient samples that contained very few numbers of CTCs had low %CV values for both HLA I and PD-L1 expression, which demonstrated the high precision of this methodology and the single-cell resolution of the read-out (Fig. 3F). The sample with the lowest number of CTCs had an average of 2 CTCs per replicate (patient 6 at week 28) with a 7% PD-L1 and 8% HLA I CV. The highest CV observed in this cohort was 11% for HLA I in patient 7 at week 21, with an average of 6 CTCs per replicate. Lower numbers of CTCs tended to have higher %CV values (Fig. 3F right panel), but none were above 20%.

### Initial clinical testing

**Fig. 4:**
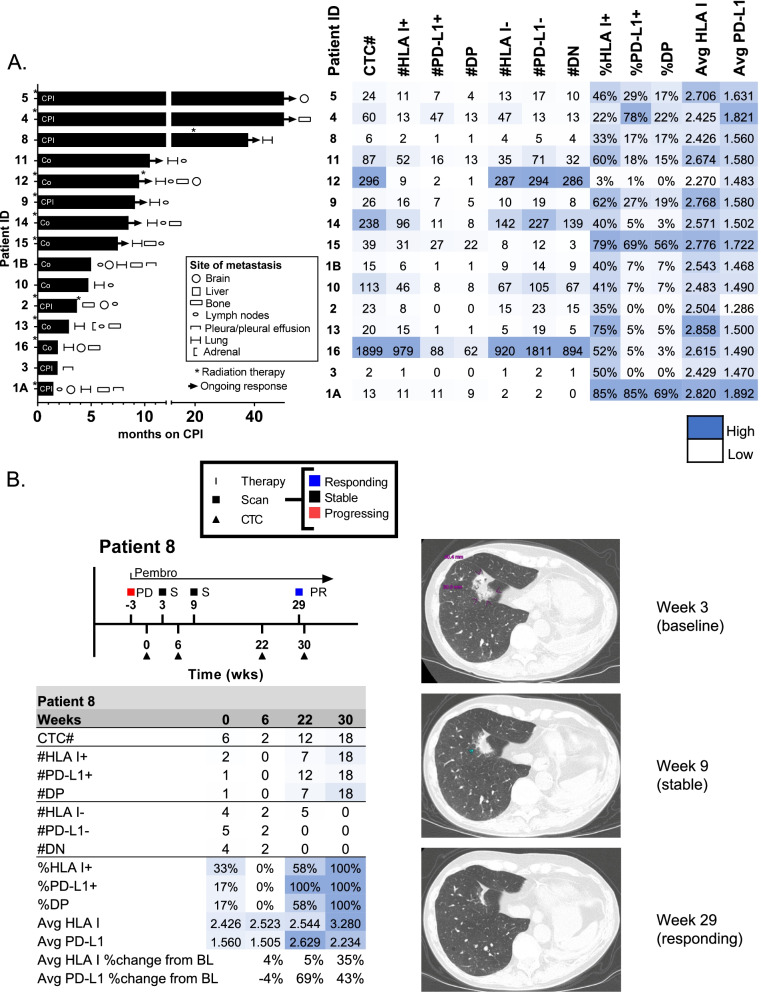
Evaluation of Clinical Correlations. **A** Swimmers’ plot of patients with baseline CTC samples, sorted by PFS. The legend indicates site(s) of metastases, asterisks indicate time of receipt of palliative radiation therapy, and arrows denote ongoing response to ICB. CPI denotes that the patient received single-agent checkpoint inhibitor therapy, and Co denotes that the patient received combination checkpoint inhibitor with chemotherapy (with exception of 1B, who received combination checkpoint inhibitor therapy with a targeted kinase inhibitor on a clinical trial). The heat map summarizes CTC parameters including enumeration of total CTCs, PD-L1-positive CTCs, and HLA I-positive CTCs. The mean fluorescence intensities of HLA I and PD-L1 expression per CTC are averaged in the heatmap. **B** Serial evaluation of CTC number and protein expression compared to radiographic assessment

Clinical outcomes of patients with Stage IV NSCLC treated with checkpoint immunotherapy (ICI), alone or combination (Co), were evaluated in parallel to CTC samples prior to initiating immunotherapy-containing treatment (Fig. 4A). This patient cohort was heterogeneous, with regard to specific treatment regimens, number of lines of therapy, receipt of radiation therapy, and sites of metastasis. CTCs were identified at baseline in all patients, with a median of 26 CTCs. Despite this heterogeneity, patients who did not respond to ICI (< 6 mo duration of response) had a lower percentage of CTCs that were positive for PD-L1 at baseline (Supplemental Fig. 5). The average expression of PD-L1 on CTCs also correlated with durable clinical benefit, with a ROC curve-pinpointed cutoff of 1.500 logMFI generating a Kaplan-Meier hazard ratio of 8.7. This data suggests that CTC phenotypic analysis provided complementary information for testing in prospective clinical trials.

Interestingly, average expression and % positivity of baseline CTC PD-L1 did not correlate with %PD-L1 positivity on tumor biopsies (Supplemental Fig. 4), nor did baseline tissue PD-L1 correlate with durable clinic benefit to ICI (Supplemental Fig. 5). The discordance between the liquid biopsy PD-L1 levels and those quantified on the solid tumor biopsy tissue did not appear to be caused by samples with low numbers of CTCs, as only 2/15 samples had fewer than 20 CTCs. All patients had HLA detection at baseline, however 2/15 baseline samples had no PD-L1-positive CTCs. 3/15 baseline samples lacked PD-L1 and HLA I positive CTCs (Fig. 4). While the cohort is insufficient to demonstrate superiority to tumor biopsies, this data identified a subpopulation of PD-L1 and HLA I positive tumor cells that associated with response to checkpoint inhibitors.

Figure 4B and Supplemental Table 1 depict longitudinal assessment of PD-L1 and HLA I expression on CTCs and demonstrate feasibility of monitoring dynamic changes over the course of therapy. Patient 8 demonstrated increasing number of total CTCs over the first 30 weeks of therapy, however 100% of these CTCs showed double-positive expression of both HLA I and PD-L1, corresponding to radiographic response on computed tomography scans. This is consistent with preliminary findings that durable clinical benefit (response lasting 6 months or greater) correlated with %PD-L1-positive or %double-positive CTCs, but not the total number of CTCs (Supplemental Fig. 5). Average PD-L1 and HLA I expression on CTCs also increased by greater than 20% from baseline during this time (Fig. 4B), exceeding the levels of analytical variability observed with this method, suggestive of true biological change. Longitudinal assessment of CTCs from three additional patients are presented in Supplemental Table 1 as hypothesis-generating examples of potential pharmacodynamic application of the results. The complexity of the CTC data highlights the richness of the dataset and the challenge of determining which parameter will provide the highest clinical value.

## Discussion

CTC enumeration has known prognostic value in NSCLC with emerging data suggesting phenotypic analysis may provide further biologic information. The evaluation of protein biomarker expression on CTCs requires fluorescence microscopy with quantitative capabilities, due to the rarity of the target cells, and the complex array of cell types that are present in blood populations. Rare CTCs are enriched from whole blood with ESP technology: concentrating ~ 1 CTC per billion blood cells down to 1 CTC per 50 blood cells, but CTCs must still be accurately identified and quantified for biomarker expression to achieve clinically useful diagnostic information. Traditional microscopy, with real-time focusing and subjective interpretation, would be too labor-intensive and variable for clinical implementation. However, quantitative systems designed for abundant sample quantities, such as flow cytometry, are ill-suited to rare cell targets where image artifacts pose a greater risk to rare-cell evaluations, requiring manual review of objects identified as CTCs.

This quantitative microscopy approach controls for the traditionally limiting workflows of microscopy and leverages the high-throughput advantages of flow cytometry. Rare cells are processed with ESP microfluidics and retained as suspensions for imaging to avoid sample loss which can occur during transfer to immobilized surfaces. Glass imaging chambers and uniform focus inverted fluorescence microscopy provide high quality imaging for cells in suspension. In combination with automated image analysis, the quantitative data generated is compatible with data analysis workflows used in flow cytometry but provides the ability to manually review every image of every cell. Using these methods, we achieved high precision biomarker quantification, along with preliminary indications of clinical utility, generating rationale for pursuing these liquid biomarkers in prospective clinical trials.

Before test systems can be formally validated within a CLIA-compliant facility, experimental approaches must first be designed that successfully evaluate and demonstrate high performance of the assay itself. Contrived test systems must be reliable enough to truly evaluate the method in question, independent of bias associated with the contrived sample types. Methods and test systems often need to be re-developed several times before they attain the reliability required for clinical implementation. This dataset demonstrates the clinical-grade performance characteristics of the ESP-quantitative microscopy approach and the ability of the contrived sample types to evaluate and monitor its performance over time. The controls in our system indicate a low coefficient of variance, well below the 20% threshold required for a CLIA-certified assay.

While clonal heterogeneity may be expected of CTC populations, test systems must still demonstrate adequate precision between replicates to be considered acceptable per CLIA standards. Cell lines represent a relatively uniform population, but CTCs from patient samples can be composed of different phenotypic, genotypic, and epigenetic types of cells. CTC heterogeneity may confound attempts to demonstrate methodological precision, where rare cell populations are inefficient at uniform distribution (i.e., stochastic variation), and summary statistics are derived from a very small numbers of cells (e.g., average biomarker expression of only a few CTCs). To confirm whether these methods would provide enough precision for patient sample evaluation, we expanded the precision assessment to include patient sample replicates. This data set suggests the use of ESP technology coupled with quantitative microscopy achieves high precision even for patient samples with very few CTCs, illustrating the potential for this method to successfully navigate the path through formal analytical validation to clinical implementation and application. The patient sample precision assessment represents a small cohort of 24 samples from 11 unique patients, and larger studies will be needed to confirm these preliminary findings, particularly for patients with very low numbers of CTCs. However, as low numbers of CTCs have been shown to correlate with better prognosis [37], this assay may prove more clinically useful for patients with higher numbers of CTCs who may see greater benefit from the identification of targeted resistance.

An additional cohort of patient samples was collected to assess the clinical utility of the assay and demonstrates the feasibility to detect these biomarkers but requires testing in larger prospective clinical trials for diagnostic validation of the preliminary clinical correlations. A limitation of our study is the heterogeneity of our patient cohort, as they have differing numbers and sites of metastases, and were treated with different therapies. Thus, this limits the ability to correlate CTC data with clinical outcomes. However, this study establishes the feasibility and potential clinical utility of multiplexed phenotypic analysis of rare CTC populations in patients with metastatic NSCLC and moves beyond enumeration as a biomarker, to potentially provide both predictive and pharmacodynamic application.

In this small patient cohort, the solid tissue biopsy results were discordant with the liquid biopsy results. However, there is significant debate in the field whether a tumor biopsy represents the phenotype of all metastatic sites, and CTCs may provide the opportunity to evaluate cancer cells shed from all sites of disease. Heterogeneity in PD-L1 expression between metastatic sites has been observed with rapid autopsy studies of patients with metastatic prostate cancer [38] and in rapid post-mortem tissue donated from patients with NSCLC [39]. While some studies have shown correlation in the expression of PD-L1 on solid tissue biopsy compared to liquid biopsy [40], several publications have demonstrated discordance [41–43]. The correlation observed between the liquid biopsy results and the survival outcomes in this cohort suggest the data may represent a biological observation, warranting larger prospective assessment.

## Conclusions

Therapies that target PD-L1 have been effective in many patients with NSCLC but improving predictive biomarkers for innate and acquired resistance remains a clinical challenge. Multiple biomarkers including PD-L1 expression on tissue biopsy, tumor mutational burden, and an inflamed gene expression score have been identified as potential biomarkers to predict response to immunotherapy. However, the landscape of treatment is rapidly expanding and now includes combination therapies including chemo-immunotherapy, treatment with multiple checkpoint inhibitors (such as combined anti-PD-L1/PD-1 and anti-CTLA4 blockade), and combining radiation therapy and immunotherapy, all approaches that require additional predictive biomarker discovery.

Solid tumor biopsy screening for the expression of PD-L1 is associated with higher clinical benefit, but recent evidence has unveiled a commonly occurring yet frequently unevaluated resistance mechanism: the downregulation of HLA I. Testing for and adjusting treatment strategies to overcome this resistance mechanism promises to improve patient outcomes for this standard of care therapeutic strategy. Additionally, repeat tumor biopsies at the time of progression is not feasible for all patients due to multiple clinical factors. Evaluating PD-L1 and HLA I in CTCs, as opposed to solid tumors, can provide the added advantage of detecting dynamic and rapid onset of this important resistance mechanism across diverse metastatic lesions. CTCs offer a minimally invasive alternative liquid biopsy to identify phenotypic attributes of the tumor, reflect disease heterogeneity between multiple metastatic sites, and can be used to monitor longitudinal changes over the course of therapy. Our approach furthers the translational development of promising technologies to advance precision immunotherapy.

## Supplementary Information

**Additional file 1: Supplemental Figure 1.** Example Distribution of Biomarker Expression. A) Each symbol represents the expression level of each individual cell identified in the final well after CTC capture and staining. Lines on graphs indicate thresholds used to define positive expression. Red symbols indicate cells identified as CTCs, and black or grey symbols represent background non-CTCs that carried over into the final well. Data represents all cells identified from one sample from patient 22. B) Autofluorescent signal detected on all cells after processing with hoechst only and C) side-by-side comparison of the same two patients with all stains included. **Supplemental Figure 2.** Assessment of Spectral Overlap. Absence of spectral overlap was confirmed by acquiring images of either compensation beads (UltraComp eBeads) or cell line cells (H358) that were stained with each of the fluorophores associated with the patient sample antibody set. Briefly, beads were stained with one of each fluorescent antibody in addition to a BV421 tag to enable automated algorithm-based image analysis. Cell line H358 cells were stained with hoechst to evaluate any spectral overlap due to the hoechst stain. Images of beads and cells were acquired with all wavelengths used in the patient sample antibody set, acquiring the wavelengths in the same order as patient sample imaging. Each symbol represents the average intensity of all beads within one image, with *n* = 3 replicate images acquired for each condition. Controls of beads with BV421 alone, beads without any fluorescent tag, and cells without hoechst were used to quantify background intensity. **Supplemental Figure 3.** Accuracy of Quantifying Positive Biomarker Expression. LNCaP and H358 cell line cells were mixed together at different ratios prior to staining and image analysis (100:0, 60:40, 40:60, 0:100), then quantified for the number of either PD-L1+ or HLA I+ cells within each condition. The number of expected positive cells was calculated by multiplying the number of each cell line in the final mixture by the frequency of positivity quantified from the pure populations. The observed number showed a linear relationship with the expected number (R^2^ = 0.9981 for PD-L1 and 0.9993 for HLA I). **Supplemental Table 1.** Serial CTC Evaluation From Multiple Patients. Serial evaluation of CTC number and protein expression compared to radiographic assessment in three additional patients. DP: Double-positive, DN: Double-negative, Nivo: Nivolumab, Atezo: Atezolizumab, Pembro: Pembrolizumab, R: Responding, S: Stable, P: Progressive Disease. **Supplemental Table 2.** Clinical Details. Patient clinical characteristics are summarized here, including age, histology, treatments rendered (including systemic therapy, surgical resection, and/or radiotherapy), pertinent genomic alterations, tissue tumor mutation burden (if known), and tissue PD-L1 expression. All patients in our cohort had a diagnosis of stage IV metastatic non-small cell lung cancer. Np indicates that the test was not performed. An asterisk in the other genomic alterations column indicates that alterations were evaluated using a limited lung-cancer specific panel – alterations in all other patients were identified using broad next-generation sequencing panels. Adeno: adenocarcinoma, NOS: not otherwise specified, N: Nivolumab, A: Atezolizumab, P: Pembrolizumab, Ch: Chemotherapy, Ch/IO: Chemoimmunotherapy, TKI: Tyrosine kinase inhibitor, XRT: radiation therapy, LN: lymph node. **Supplemental Figure 4.** Correlation Between Liquid and Solid Biopsy. CTC characterization at baseline compared to the tissue biopsy results in our patient cohort. Percentage of PD-L1 positive tumor cells on tissue biopsy were compared to percentage of PD-L1 positive CTCs (A) or the log mean fluorescence intensity of PD-L1 on CTCs (B). There is no correlation between tissue and CTC PD-L1 expression on either parameter. The number of CTCs detected in each patient sample was labeled next to each symbol. **Supplemental Figure 5.** Clinical Correlation of Tissue or Baseline CTC Evaluation on Prolonged Response. Solid tumor biopsy PD-L1 levels and baseline liquid biopsy PD-L1 and HLA I parameters were compared for the ability to predict progression-free survival (PFS) of less than or greater than 6 months. T-tests (all assuming normal distribution except total #CTC which did not) were used to assess the ability of each of the different characteristics to differentiate between patients with shorter or longer PFS (> 6 months). Kaplan-Meier curves were generated for tissue PD-L1, with a cutoff of 1% positivity, and for average CTC PD-L1, with a cutoff of 1.5.

## Data Availability

The datasets analyzed during the current study are available from the corresponding author on reasonable request.

## Abbreviations

PD-L1: Programmed death ligand 1
HLA I: Human leukocyte antigen class I
NSCLC: Non-small cell lung cancer
CTCs: Circulating tumor cells
OS: Overall survival
LOH: Loss of heterozygosity
ESP: Exclusion-based sample preparation
CLIA: Clinical laboratory improvement amendments
ATCC: American type culture collection
RPMI: Roswell park memorial institute
TRIP: Translational research initiatives in pathology laboratory
ELISA: Enzyme linked immunosorbent assay
RT: Room temperature
IRB: Internal review board
PBMC: Peripheral blood mononuclear cells
MACS: Magnetic assisted cell sorting
PMP: Paramagnetic particle
FS: Focus surface
PFS: Perfect focus system
MFI: Mean fluorescence intensity
CV: Coefficient of variation
ROC: Receiver operating characteristic
ICI: Immune checkpoint inhibitor
Co: Combination
