# Supplementary material for: Analytical validation and initial clinical testing of quantitative microscopic evaluation for PD-L1 and HLA I expression on circulating tumor cells from patients with non-small cell lung cancer
Source: Biomark Res. 2022 Apr 25;10:26. doi: 10.1186/s40364-022-00370-8 (PMC9040226; doi:10.1186/s40364-022-00370-8)
Supplement: Supplementary file 1 — Additional file 1: Supplemental Figure 1. Example Distribution of Biomarker Expression. A) Each symbol represents the expression level of each individual cell identified in the final well after CTC capture and staining. Lines on graphs indicate thresholds used to define positive expression. Red symbols indicate cells identified as CTCs, and black or grey symbols represent background non-CTCs that carried over into the final well. Data represents all cells identified from one sample from patient 22. B) Autofluorescent signal detected on all cells after processing with hoechst only and C) side-by-side comparison of the same two patients with all stains included. Supplemental Figure 2. Assessment of Spectral Overlap. Absence of spectral overlap was confirmed by acquiring images of either compensation beads (UltraComp eBeads) or cell line cells (H358) that were stained with each of the fluorophores associated with the patient sample antibody set. Briefly, beads were stained with one of each fluorescent antibody in addition to a BV421 tag to enable automated algorithm-based image analysis. Cell line H358 cells were stained with hoechst to evaluate any spectral overlap due to the hoechst stain. Images of beads and cells were acquired with all wavelengths used in the patient sample antibody set, acquiring the wavelengths in the same order as patient sample imaging. Each symbol represents the average intensity of all beads within one image, with n = 3 replicate images acquired for each condition. Controls of beads with BV421 alone, beads without any fluorescent tag, and cells without hoechst were used to quantify background intensity. Supplemental Figure 3. Accuracy of Quantifying Positive Biomarker Expression. LNCaP and H358 cell line cells were mixed together at different ratios prior to staining and image analysis (100:0, 60:40, 40:60, 0:100), then quantified for the number of either PD-L1+ or HLA I+ cells within each condition. The number of expected positive cells was [file 40364_2022_370_MOESM1_ESM.pdf]

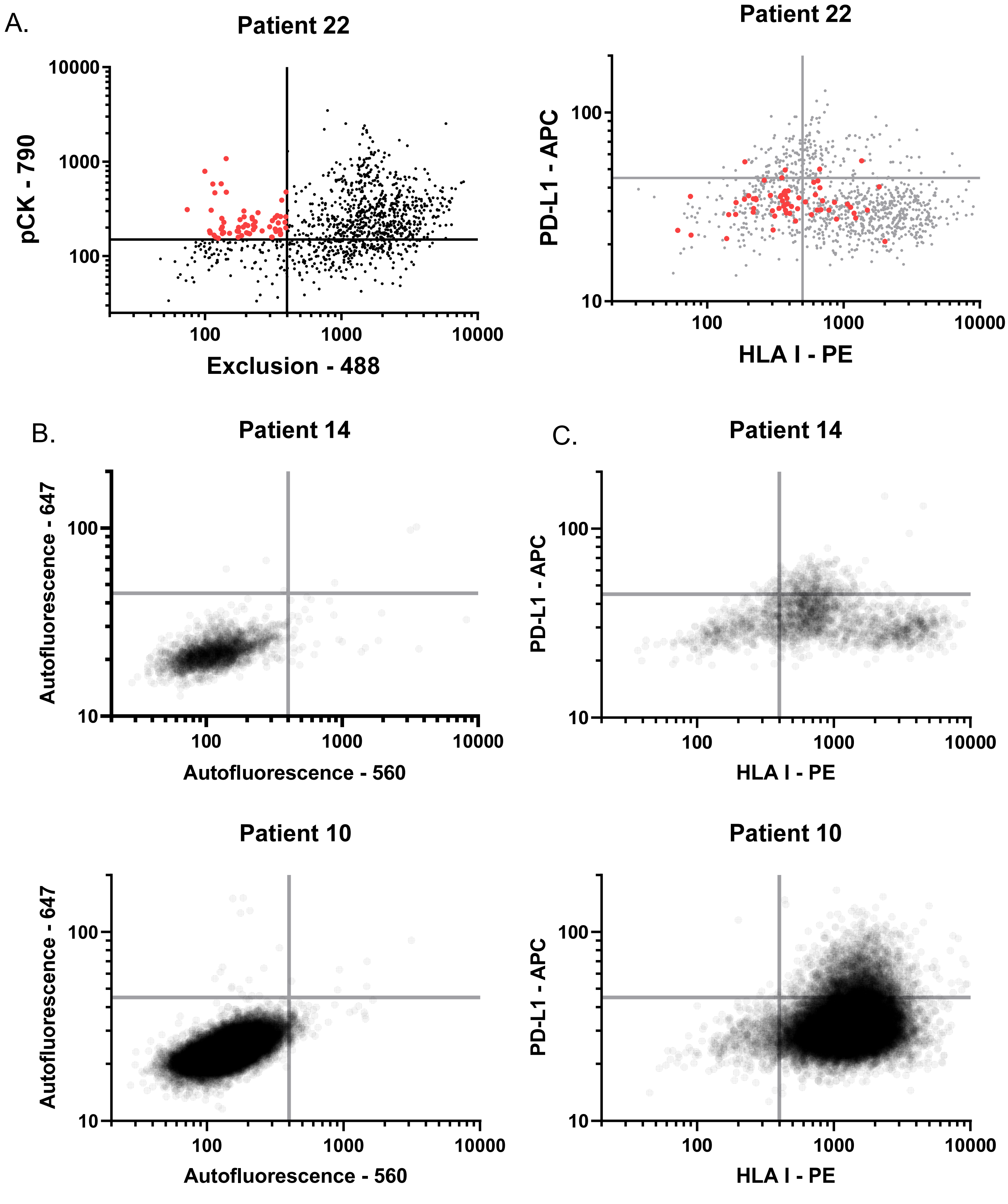

Supplemental Figure 1

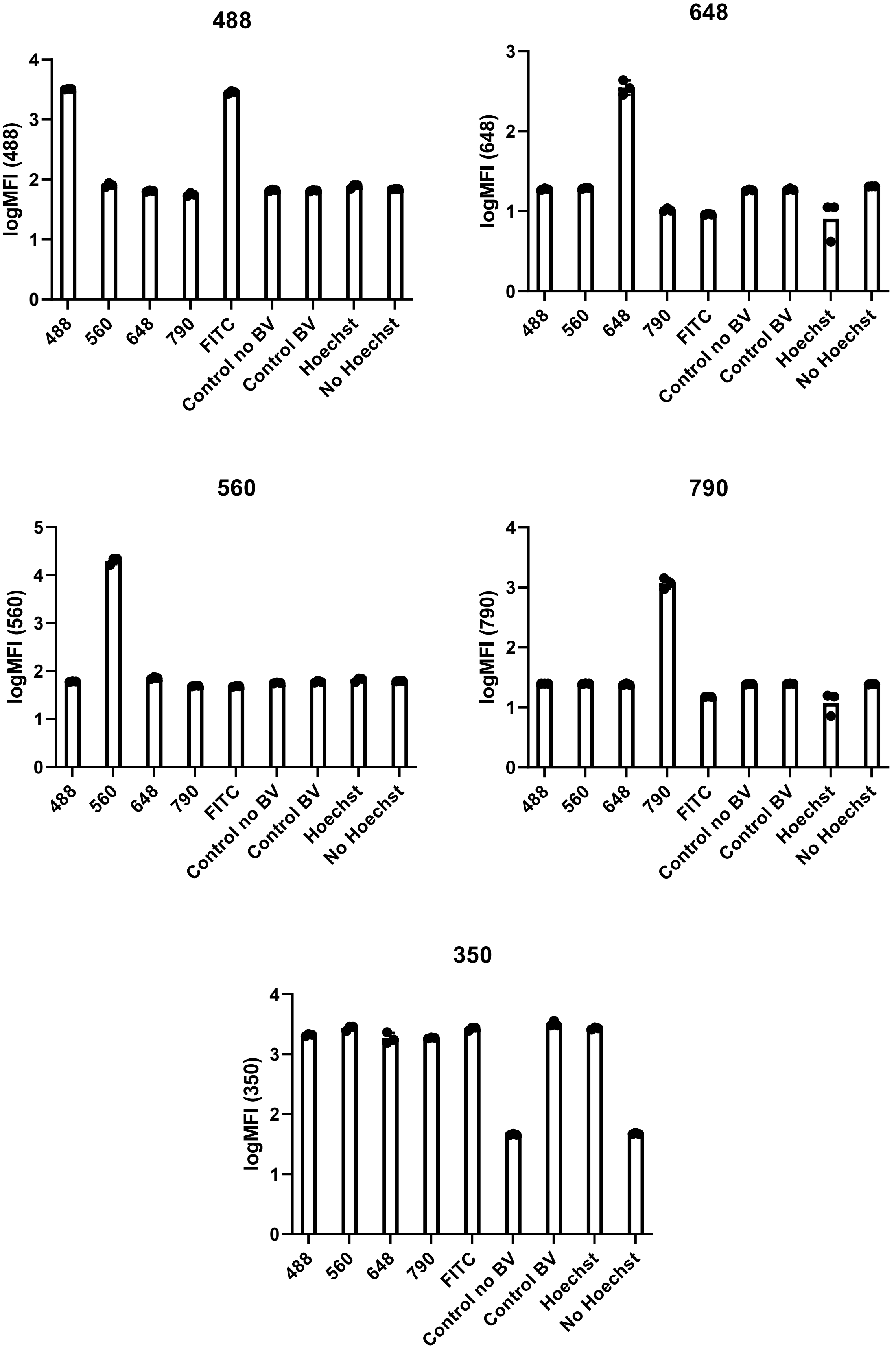

Supplemental Figure 2

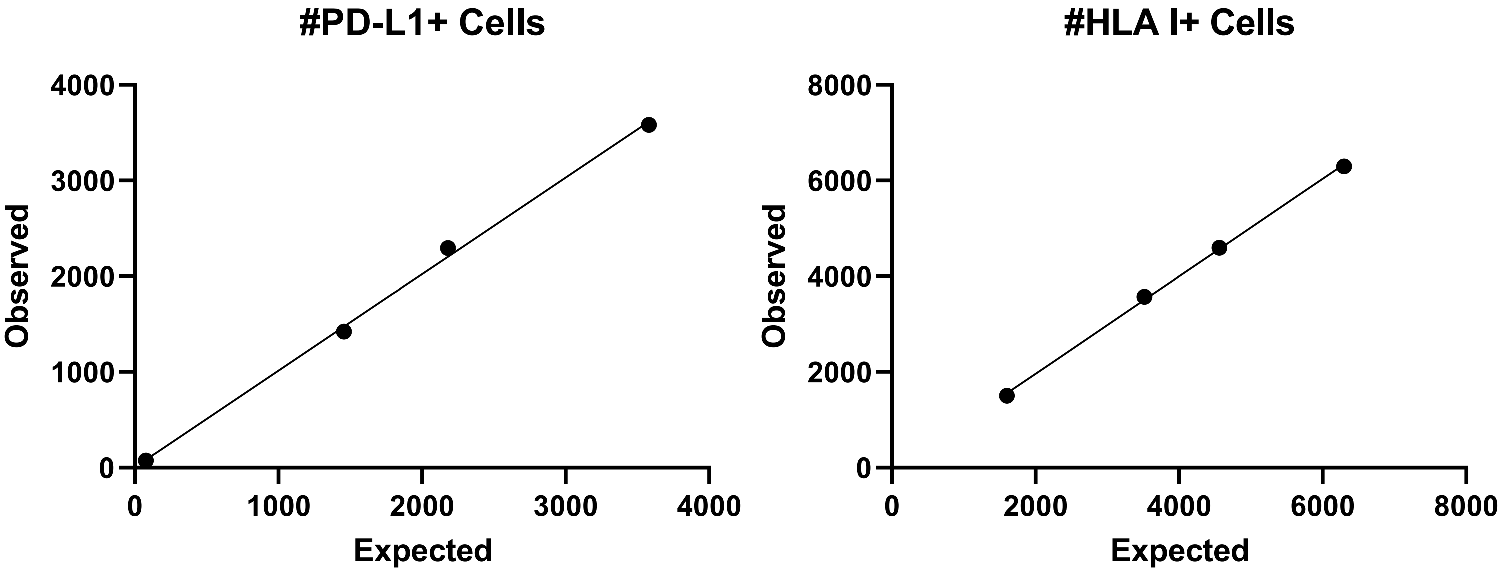

Supplemental Figure 3

Max	Min	Patient ID	1						4							7								
Max	Min	**Weeks**	**0**	**22**	**28**	**35**	**41**	**47**	**0**	**6**	**15**	**34**	**40**	**57**	**69**	**0**	**16**	**21**	**33**	**39**	**45**	**51**	**64**	**69**
**219**	0	CTC#																						
**101**	2	#HLA I+																		-			-	
**215**	0	#PD-L1+																		-			-	
**18**	0	#DP																		-			-	
**201**	0	#HLA I-																		-			-	
**141**	2	#PD-L1-																		-			-	
**119**	0	#DN																		-			-	
**100%**	8%	%HLA I+																		-			-	
**98%**	0%	%PD-L1+																		-			-	
**69%**	0%	%DP																		-			-	
**3.04**	2.15	Avg HLA I																		-			-	
**2.15**	1.30	Avg PD-L1																		-			-	
		Avg HLA I change from BL		-7%	-16%	-29%	-8%	-20%		-3%	19%	4%	7%	5%	7%		28%	37%	14%	-	17%	7%	-	10%
		Avg PD-L1 change from BL		31%	2%	7%	3%	4%		18%	-14%	-15%	-15%	-17%	-29%		2%	2%	-3%	-	2%	-3%	-	-7%
		Prior Scan Reading	P	NA	NA	NA	S	P	NA	S	S	S	S	S	S	NA	R	R	S	S	S	S	S	S
		Weeks Since Prior Scan	22	-	-	-	4	2	-	3	6	6	6	5	7	-	3	9	3	0	6	3	15	2
		Next Scan Reading	S	NA	NA	S	NA	P	S	S	S	S	S	S	S	R	S	S	S	S	S	S	S	S
		Weeks Before Next Scan	0	-	-	-2	-	-2	-2	-3	-7	0	0	-5	-12	-3	-8	-2	-6	-9	-3	-16	-3	-3
		Weeks Since XRT	-	5	-	-	-	-	8	-	-	-	-	-	-	-	-	-	-	-	-	-	-	-
		Weeks on Therapy:	21	3	3	6	12	4	3	9	18	37	43	60	6	7	22	28	40	46	52	58	70	76
		Baseline			X			X							X									
		Nab-Paclitaxel	X																					
		Pembro		X												X	X	X	X	X	X	X	X	X
		Sitra/Nivo				X	X																	
		Atezo							X	X	X	X	X	X										

Supplemental Table 1

ID	Age	Sex	Histology	KRAS mutation	Other Genomic Alterations	TMB (mut/ Mb)	PD-L1 Biopsy (TPS)	#Lines systemic therapy	P	N	A	Ch	Ch/IO	TKI	XRT	Sites of Mets
1	60	F	adeno	G12S	None*	np	np	6	x	x		x			x	lung, pleura, bone, LN, brain
2	86	M	adeno	G12C	None*	np	100%	2	x			x			x	LN, bone, brain
3	66	F	adeno	G12A	CTNNB1	np	<1%	2			x	x				pleura with malignant effusion
4	61	M	adeno	unknown	inadequate tissue	np	20%	1			x				x	bone
5	63	M	adeno	no	None*	np	<1%	1			x				x	brain
6	76	F	adeno	no	None*	np	100%	1	x							Lung
7	66	M	squamous	np	np	np	80%	1	x							LN, lung
8	74	F	adeno	no	KRAS amplified (8), CDKN2A	np	98%	2	x			x				lung
9	73	F	adeno	no	TP53, CHEK2	np	80-90%	1	x							lung, LN
10	81	M	adeno	no	IDH2, STK11, ARID1A	3.2	<1%	1					x			lung, LN
11	64	M	squamous	np	np	np	50%	1					x			lung, LN
12	68	M	NSCLC NOS	G12F	ATM, SMAD4	8	>99%	1					x		x	lung, LN, bone, brain
13	83	M	adeno	no	TP53, STK11, RIT1	np	<1%	1					x			lung, adrenal, bone, LN
14	64	F	adeno	G12D	TP53, ATRX	21.9	10%	1					x		x	lung, bone, LN
15	56	M	adeno	G12V	STK11, ARID1A, KEAP1	23.89	1%	1					x		x	lung, bone, LN
16	69	F	adeno	G12C	TP53, SMAD4 splice site SNV	np	np	1					x		x	lung, brain, bone
17	72	F	squamous	G12V	TP53	np	90%	3	x						x	liver, adrenal, brain, bone, LN
18	67	F	adeno	no	ALK fusion*	np	np	2				x		x	x	brain
19	80	F	adeno	no	EGFR 19 del*, T790M	np	np	2						x	x	lung, bone, LN
20	46	M	adeno	no	ALK fusion*	np	np	1						x		brain, pleura
21	82	F	adeno	no	EGFR*	np	np	2						x	x	bone, brain
22	60	F	adeno	no	ALK fusion*	np	1-5%	1						x	x	bone, brain, pleura, periatrial

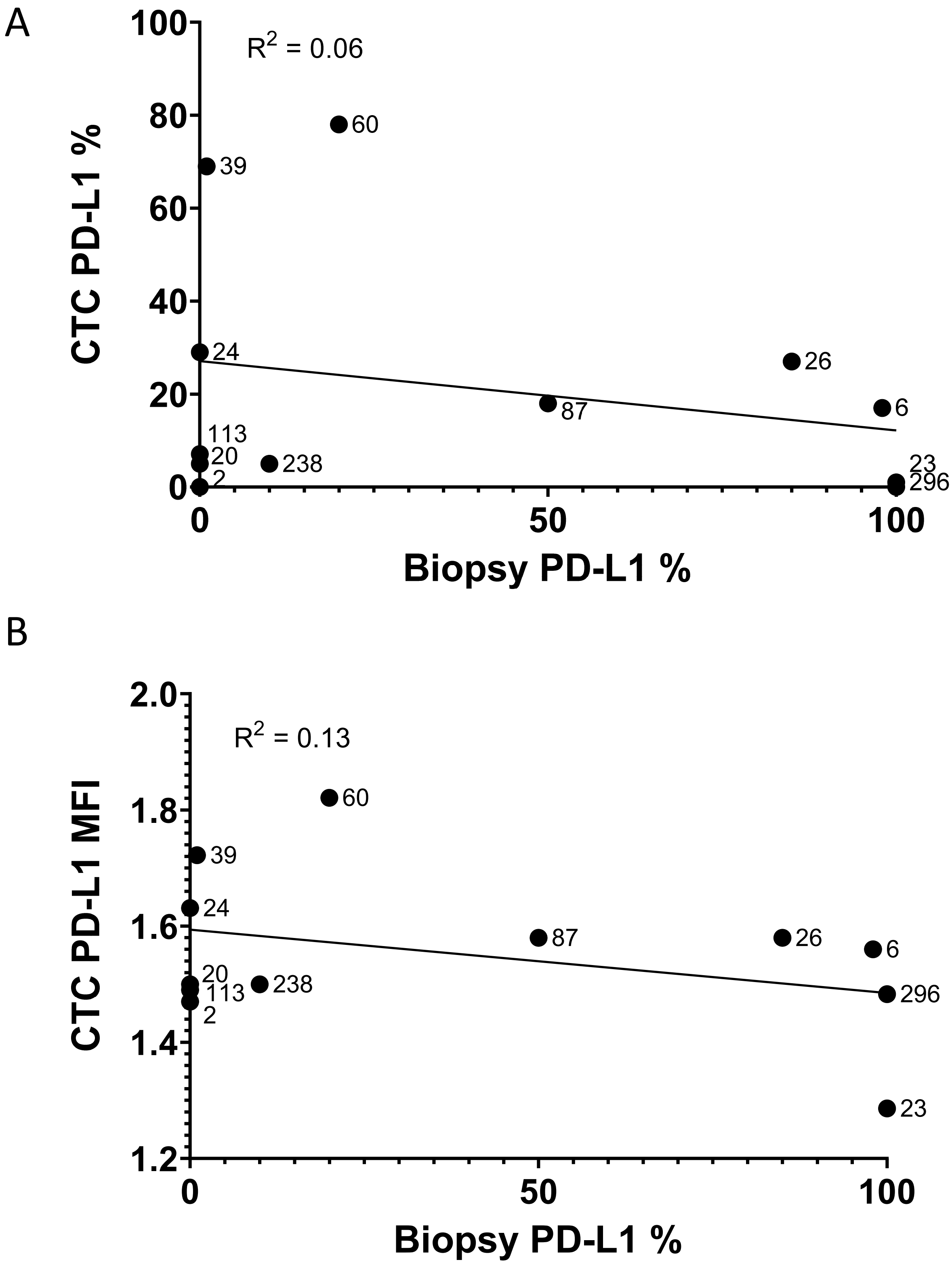

Supplemental Figure 4

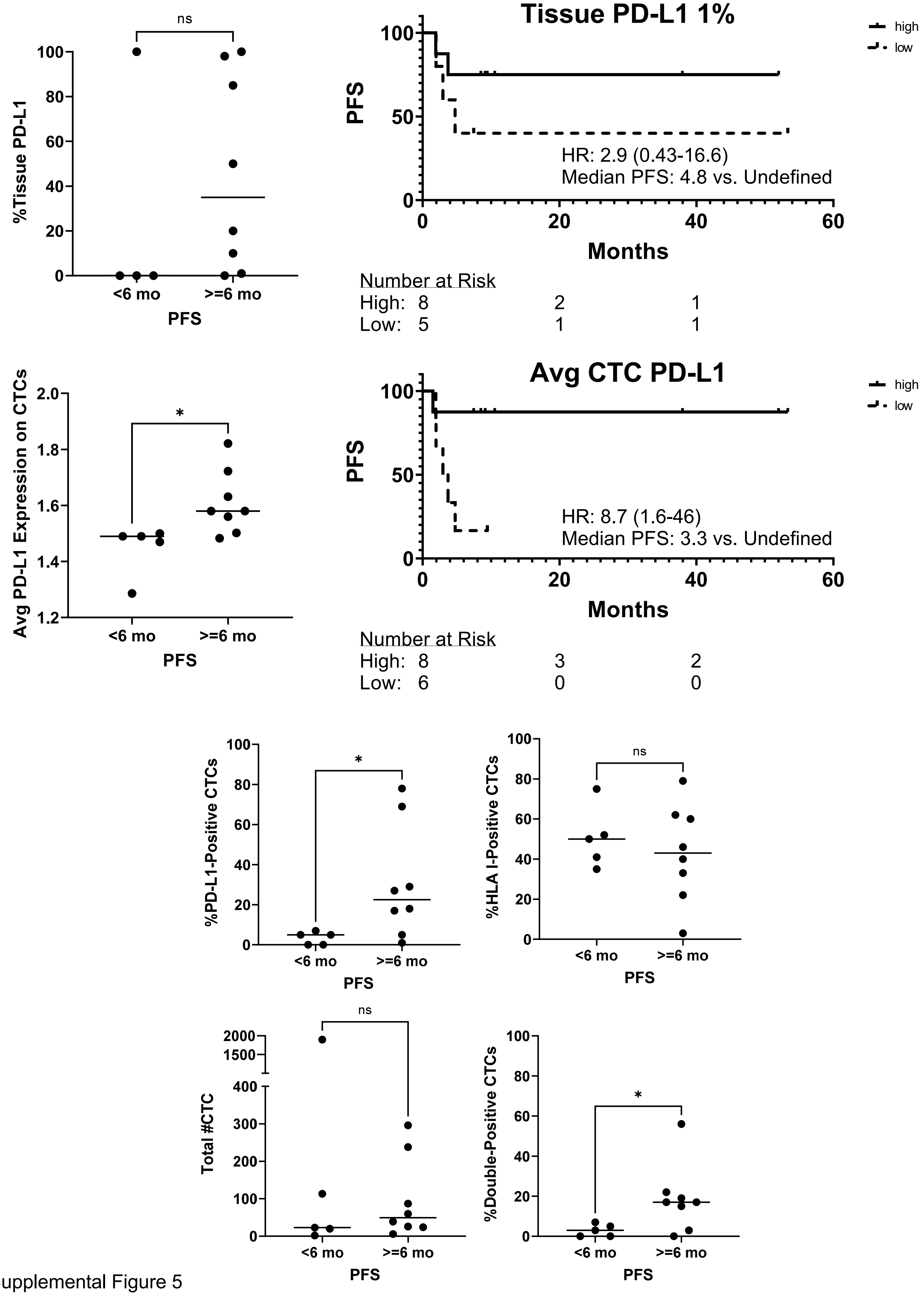

Supplemental Figure 5
